# Triterpenoids and Polysaccharide Fractions of *Ganoderma tsugae* Exert Different Effects on Antiallergic Activities

**DOI:** 10.1155/2015/754836

**Published:** 2015-04-19

**Authors:** Miaw-Ling Chen, Chia-Chien Hsieh, Bor-Luen Chiang, Bi-Fong Lin

**Affiliations:** ^1^Department of Nutrition and Health Sciences, College of Health Sciences, Chang Jung Christian University, No. 1 Changda Road, Gueiren District, Tainan 71101, Taiwan; ^2^Department of Human Development and Family Studies, College of Education, National Taiwan Normal University, No. 162 Hepting East Road, Section 1, Taipei 10610, Taiwan; ^3^Graduate Institute of Clinical Medicine, College of Medicine, National Taiwan University, No. 7 Chung San South Road, Taipei 10002, Taiwan; ^4^Department of Biochemical Science and Technology, College of Life Science, National Taiwan University, No. 1 Roosevelt Road, Section 4, Taipei 10617, Taiwan

## Abstract

This study was to investigate antiallergic effects of triterpenoids (Gt-TRE) and polysaccharide (Gt-PS) extracts from *Ganoderma tsugae*, using mast cell line RBL-2H3, T cell line EL4, primary T cells, and transfected RAW264.7 macrophage cells. The results showed that histamine secreted from activated RBL-2H3 mast cells was significantly suppressed by Gt-TRE but not Gt-PS. Interleukin- (IL-) 4 secreted from activated EL4 cells was significantly suppressed by Gt-TRE but not Gt-PS. Further primary CD4^+^ T cells cultures also confirmed that Gt-TRE (5 ~ 50 *µ*g/mL) significantly suppressed Th2 cytokines IL-4 and IL-5 secretions but had no effect on Th1 cytokines IL-2 and interferon (IFN)-*γ*. Gt-PS did not affect IL-4 and IL-5 secretions until higher doses (400, 500 *µ*g/mL) and significantly suppressed IFN*γ* secretions but enhanced IL-2 at these high doses. The reporter gene assay indicated that Gt-TRE inhibited but Gt-PS enhanced the transcriptional activity of NF-*κ*B in activated transfected RAW264.7 cells and transfected EL4 cells. IL-4 secreted by this transfected EL-4 cells was also significantly decreased by Gt-TRE but not by Gt-PS, suggesting that these two fractions may exert different effects on NF-*κ*B related cytokines expression. These data suggested that triterpenoids fraction of *Ganoderma tsugae* might be the main constituents to alleviate allergic asthma.

## 1. Introduction

The worldwide increase in prevalence of allergic diseases such as asthma [1, 2] poses a significant health problem and a demand for drug/diet therapy. Allergic asthma is characterized by histamine secretion by mast cell, bronchial hyperresponsiveness, and airway inflammation by accumulation of eosinophils, lymphocytes and mast cells, and higher serum IgE levels [3]. The allergic immune responses in asthma arise from an imbalance of helper T (Th) cells. Th1 cells and their cytokines IL-2 and IFN*γ* enhance Th1 generation and inhibit Th2 function, whereas Th2 cells and their cytokines inhibit Th1 generation and rise allergic responses. IL-4 secreted by Th2 cells stimulates B cells class switch to produce allergic immunoglobulin (Ig) E and promote neutrophil- and eosinophil-infiltrated inflammation. These cells were also activated by another Th2 cytokine IL-5. Therefore, downregulating Th2 cell differentiation by cytokine administration is frequently used as a therapy for allergic diseases [4]. Several studies indicated dietary factors, such as frying oil, adlay, and andrographolide from* Andrographis paniculata* suppressed Th2 immune responses in the Th2-skewed ovalbumin- (OVA-) sensitized BALB/c mice [5–7], and polysaccharide from fruiting bodies of* Ganoderma lucidum* has been shown to mediate cytokines production [8]. Therefore, the immunomodulatory effects of* G. tsugae* rose our interest.

The polypore genus* Ganoderma* had been widely used for Chinese medicine in Asian countries for a long time. It has been known for many biological activities of* Ganoderma*, such as antitumor, immunoregulation, hepatoprotection, anticholesterol synthesis and anti-inflammation [9–14]. Although the major active ingredients are polysaccharides, triterpenoids and proteins, polysaccharides are most studied [15]. The polysaccharides with immunity enhancement effects have been isolated from the water extract of* G. lucidum* mycelia and fruiting bodies [16]. It has been demonstrated that polysaccharide extracts of* G. lucidum* exert immunomodulating activities by inducing cytokine expression* via* TLR4 signaling pathways, activation of dendritic cells, and innate immunity by NF-*κ*B pathways [17–19]. The different ingredients may exert diverse bioactive functions [20]. Although recent review articles also summarized the health benefits of* Ganoderma*, especially triterpenoids [21], the immunomodulatory effects of triterpenoids still need to be clarified.

*G. lucidum* is the most studied species of the* Ganoderma*. However, another species of medicinal mushrooms,* G. tsugae*, is most widely cultivated in Taiwan and used as functional foods. Our previous study showed that* G. tsugae* supplementation significantly enhanced Th1/Th2 balance in the Th2-skewed OVA-sensitized and challenged BALB/c mice as an allergic inflammation model [22]. Further study showed that* G. tsugae* supplementation significantly alleviated histamine, prostaglandin (PGE) 2 and eotaxin, a protein that can activate eosinophils and airway hyperresponsiveness, levels in bronchoalveolar lavage fluid (BALF) in OVA-sensitized allergic BALB/c mice [23]. However, the eosinophils in BALF and Th2 cytokines IL-4 and IL-5 and were not significantly suppressed by* G. tsugae *[22, 23]. Since early* in vitro* study suggested that triterpenoids suppressed histamine release from mast cells [24], our study further demonstrated that triterpenoids fraction from* G. tsugae* significantly inhibited eosinophils, IL-4, IL-5, PGE2, eotaxin levels, and thus airway hyperactivity [25].

There are studies that indicated that polysaccharide component promotes Th1 immune responses [18, 26]. Therefore, whether histamine or IL-4 production was affected by polysaccharide or triterpenoids of* G. tsugae* were investigated in this study using a mast cell line and a murine T cell line. To further investigate whether cytokine productions were affected by polysaccharide or triterpenoids* via* T cell polarization, the primary CD4^+^ T cells isolated from the DO11.10 transgenic mice were used in this study. The DO11.10 transgenic mice with high percentage of OVA peptide-specific *αβ* T cell receptor can specifically respond to OVA stimulation [27] and it is a useful model for investigating the association between allergic disease and T cells. In addition, since inhibition of* in vitro* NF-*κ*B transactivation activity was shown in accordance with* in vivo* anti-inflammation [28] and its association with T cells and dendritic cells activations [29], both RAW264.7 macrophage cell line and EL-4 murine T cell line were transfected with NF-*κ*B driven reporter plasmid to determine the actions of polysaccharide or triterpenoids of* G. tsugae*. In this study, we provide the first comparison between the polysaccharide and triterpenoid extracts of* Ganoderma tsugae* on Th2 responses and its possible mechanism of NF-*κ*B transactivation.

## 2. Materials and Methods

### 2.1. Materials and Chemicals

The polysaccharide and triterpenoid extracts were kindly provided by Double Crane Group (Yung-Kien Industry Corp., Taiwan). The fruiting bodies of* Ganoderma tsugae* YK-01 were fractionated into a polysaccharide fraction (Gt-PS) (alcohol-insoluble) as described previously [30], and an alcohol-soluble fraction. The alcohol-soluble fraction was extracted with acidic ethyl acetate to yield 4.2% triterpenoid rich extracts (Gt-TRE) as previously studied [31]. The purity of the Gt-TRE was identified by reverse-phase HPLC methods to be 38% and contained nine major peaks identified as ganoderic acids (ganoderic acids A, B, C, C5, C6, D, E, and G, and ganodermic acid D), reported previously by D.-H. Chen and W. K.-D. Chen [32].

### 2.2. Cell Lines Culture and Treatment

Cell lines used in this study were purchased from Food Industry Research and Development Institute (Taiwan). EL4 murine T cells (ATCC no. TIB-39) were grown in Dulbecco's modified Eagle's medium (DMEM, Gibco, Grand Island, NY) plus 10% FBS (Gibco) at 37°C, 5% CO_2_. EL4 T cells were cultured with Gt-TRE or Gt-PS and stimulated with 50 ng/mL phorbol 12-myristate 13-acetate (PMA, Sigma, St Louis, MO) plus 1 *μ*g/mL ionomycin (Sigma) for 24 hours, and the supernatants were collected for IL-2 and IL-4 analysis.

RBL-2H3 mast cells (ATCC no. CRL-2256) were grown in minimum essential medium eagle (MEM, Gibco) plus 15% fetal bovine serum (FBS, Gibco), 1 mM sodium pyruvate, 1.5 g/L sodium bicarbonate, and 0.1 mM nonessential amino acid. RBL-2H3 mast cells were pretreated with various doses of Gt-TRE or Gt-PS for 24 hrs and stimulated with ionomycin (500 ng/mL) for 5 hrs. After stimulation, supernatants were collected for histamine concentration analysis.

RAW264.7 macrophage cells (ATCC no. TIB-71) were grown in DMEM plus 10% FBS at 37°C, 5% CO_2_. Cells were transfected with NF-*κ*B responsive luciferase reporter plasmids, which are described in NF-*κ*B responsive reporter assay section.

### 2.3. Determination of Histamine Release

The histamine level was determined by Histamine-ELISA kit (IBL Hamburg, Germany). The procedure was according to manufacturer's instructions. In brief, the culture supernatants and plasma standards were acylated with acylation reagent. Then, aliquots of 50 *μ*L acylated sample and standards were loaded into the 96-microplate wells, and then 50 *μ*L enzyme conjugate and 50 *μ*L antiserum were added to each well. The plate was shaken carefully for 3 hours on an orbital shaker at room temperature. The plate was washed four times and 200 *μ*L tetramethylbenzidine (TMB) substrate solution was added to each well for 30 min and stopped by 100 *μ*L of TMB stop solution. The optical density was measured with a microplate autoreader at 450 nm. The histamine concentrations were determined according to the standard curve.

### 2.4. Isolated and Cultured OVA-Specific Naïve CD4^+^ T Cells

The CD4^+^T cells were isolated from spleen of BALB/c DO11.10 OVA-specific T cell receptor transgenic mice with SpinSet kit (>95% CD4^+^ purity; StemCell Technologies, Vancouver, BC, Canada). The CD4^+^ T cells (5 × 10^5^ cells/mL) were cultured in the presence of irradiated (2700 rad, 540 sec) antigen presenting cells (APC) at 2 × 10^6^ cells/mL in a final volume of 1 mL with 1 *μ*g/mL OVA_323–339_ peptide and various concentrations of Gt-TRE or Gt-PS. After 72-hour incubation for naïve CD4^+^ T cells differentiation, supernatants were collected and stored at −80°C until cytokines analysis. The IL-2 and IFN*γ* levels in the supernatants were the indicators for Th1 response, whereas IL-4 and IL-5 levels were the indicators for Th2 responses. The data is present as the relative cytokine production to the control cells without Gt-TRE or Gt-PS treatment in each experiment.

### 2.5. Determination of Cytokines Production

The cytokine levels in culture supernatants were measured by sandwich ELISA methods. Briefly, the anticytokine antibody, including purified rat anti-mouse cytokine monoclonal antibodies IL-2, IFN*γ*, IL-4, and IL-5 (PharMingen, San Diego, CA), were coated in the 96-well plates (Nunc, Roskilde, Denmark). After overnight incubation at 4°C and blocking with PBS containing 1% BSA for 30 min, the samples and standards (recombinant mouse cytokines, PharMingen) were added to the 96-well plates for 2 hours of incubation. The biotin-conjugated antibodies (biotinylated rat anti-mouse cytokine monoclonal antibodies, PharMingen) were added and incubated. After washing, the streptavidin-conjugated peroxidase (Thermo Fisher Scientific Inc., Rockford, IL) was added for 1 hour. The substrates, 2,2′-azino-bis-3-ethyl-benzthiazoline-6-sulfonic acid (ABTS, Sigma), were added to each well for 20 min to develop color. The plates were read in a microplate autoreader (Microplate autoreader; Bio-Tek Instrument, Inc. Winooski, VT) at 405 nm. The detection limits for IL-2, IFN*γ*, and IL-5 are 75 pg/mL and 3.9 pg/mL for IL-4.

### 2.6. NF-*κ*B-Dependent Luciferase Activity Assay

The NF-*κ*B-promoted luciferase reporter plasmid, 3x-*κ*B-tk-luc [33], had three copies of an NF-*κ*B binding site in the upstream thymidine kinase (tk) promoter and luciferase reporter gene in the pGL2 vector (Mock) was used to assay the activity of NF-*κ*B transactivation as described previously [34]. Plasmids were grown in the JM109 strain of* E. coli* in Luria-Bertani medium containing 100 *μ*g/mL ampicillin (Sigma), and plasmid DNA preparations were performed by the QIAGEN plasmid midi kit (QIAGEN, Hilden, Germany).

The 3x-*κ*B-tk-luciferase and Renilla luciferase reporter plasmid pRL-tk-luciferase plasmids were introduced into RAW264.7 cells or EL4 T cells by liposome-mediated method. Transfection was performed in 24-well plates using Lipofectamine 2000 reagent (Invitrogen, Carlsbad, CA) according to the manufacturers' instructions. Briefly, cells were grown in 24-well plates and transfected with the appropriate vector, 0.8 *μ*g 3x-*κ*B-tk-luciferase and 0.4 *μ*g pRL-tk-luciferase (2 : 1), in serum-free OPTI-MEM medium (Gibco BRL, Gaithersburg, MD) containing 2 *μ*L Lipofectamine 2000. After appropriate transfection times, 5 hrs for RAW264.7 cells and 24 hrs for EL4 T cells, the transfection mix was removed and replaced with complete medium. Cells were then either treated with Gt-TRE or Gt-PS or stimulated with mitogens, LPS plus IFN*γ* for RAW264.7 cells for 8 hrs, and PMA plus ionomycin for EL4 T cells for 24 hrs, respectively. The supernatants were collected for cytokine determination and the cells were for luciferase activity analysis.

Luciferase activity was determined using the Dual-Glo Luciferase assay system, according to the manufacturer's instructions from Promega (Madison, WI). Light emission was measured in a luminescence microplate counter (Wallac Victor-2 Perkin Elmer, Norwalk, MT). The efficiency of transfection, as determined by* Renilla *luciferase activity in the lysate by cotransfection with pRL-tk constructs, was used to normalize the activity of* firefly* luciferase [35]. Results were expressed as relative luciferase activity corrected for the differences in active efficiency by nonstimulated cells.

### 2.7. Statistical Analysis

The significance of difference between treatment and control groups was analyzed statistically by Student's *t*-test of the SAS program system (SAS/STATA version 8.2; SAS Institute, Cary, NC), in order to show the effect of each fraction's antiallergic properties. Differences were considered to be significant if *P* was <0.05.

## 3. Results

### 3.1. Triterpenoids, Not Polysaccharides, Suppressed Histamine Release from Mast Cells

**Figure 1:**
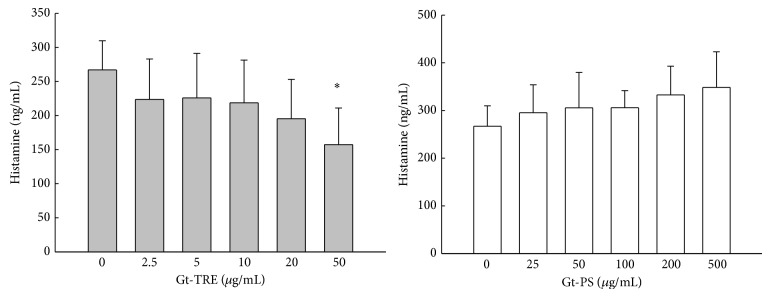
The histamine secretion from ionomycin-stimulated RBL-2H3 mast cells cultured with different doses of triterpenoids and polysaccharide extracts from* G. tsugae*. The RBL-2H3 mast cells (5 × 10^4^ cells/mL) were pretreated with Gt-TRE or Gt-PS extracts from* G. tsugae* for 24 hr, and then stimulated with 500 ng/mL ionomycin for 5 hr. The supernatants were collected and assayed by histamine ELISA kit. The data are representative for three independent experiments. ^∗^ *P* < 0.05 as compared to the 0 *μ*g/mL control cells.

Histamine is an important mediator of allergic disease. To evaluate whether triterpenoid-rich extracts or polysaccharide fractions of* Ganoderma tsugae* could inhibit the histamine secretion on allergic responses [23], histamine release from ionomycin-stimulated RBL-2H3 mast cells cultured with Gt-TRE or Gt-PS was investigated. The results as shown in Figure 1 indicated different effects of Gt-TRE and Gt-PS on histamine productions. Histamine levels were slightly decreased with increased doses of Gt-TRE and significantly suppressed by Gt-TRE at 50 *μ*g/mL. On the contrary, histamine productions were not significantly affected by Gt-PS, though they were tended to increase.

### 3.2. Triterpenoids Decreased IL-4 Production from EL4 T Cells

**Figure 2:**
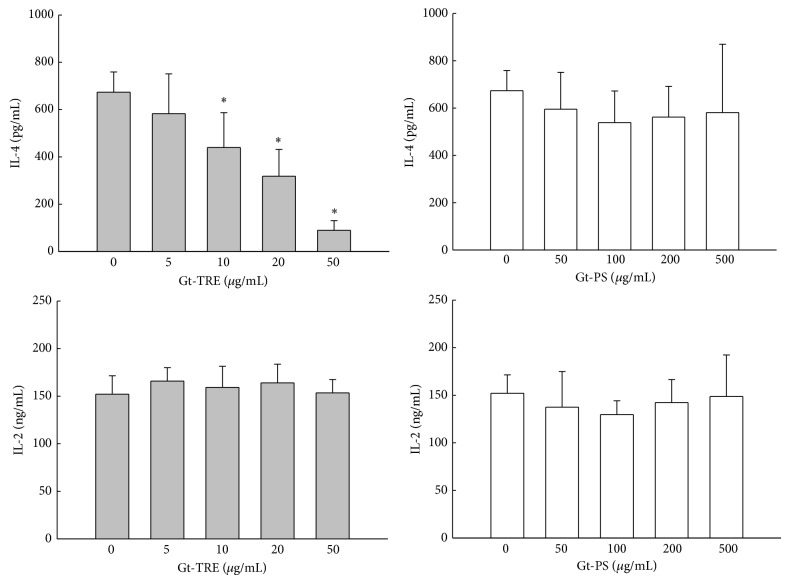
The IL-4 and IL-2 productions of activated EL4 T cells cultured with different doses of triterpenoids and polysaccharide extracts from* G. tsugae*. EL4 murine T cells (2 × 10^5^ cells/mL) were cultured with Gt-TRE or Gt-PS extracts from* G. tsugae* and activated by 50 ng/mL PMA plus 1 *μ*g/mL ionomycin for 24 hrs. The supernatants were collected and cytokine levels were determined by ELISA method. The data are representative for five independent experiments. ^∗^ *P* < 0.05 as compared to the 0 *μ*g/mL control cells.

In order to investigate the triterpenoids and polysaccharides on cytokines, IL-2 and IL-4, productions by activated murine EL4 T cells were cultured with Gt-TRE or Gt-PS (Figure 2). Under PMA plus ionomycin stimulation, Th1 cytokine IL-2 productions from EL4 T cell were not affected by Gt-PS and Gt-TRE treatment. On the other hand, IL-4 productions were decreased in a dose-dependent manner by Gt-TRE treatment but not by Gt-PS treatment. These data suggested that triterpenoid extracts of* G. tsugae* are the main constituent to suppress Th2 cytokine production.

### 3.3. Triterpenoids Decreased Th2 Cell Development and Th2 Cytokine Production

**Figure 3:**
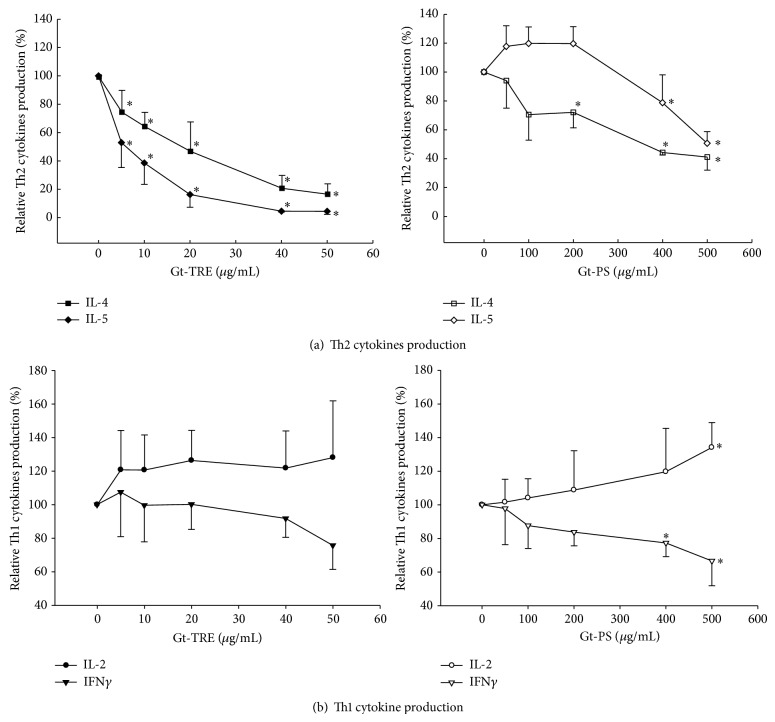
The relative Th2 cytokines (a) and Th1 cytokines (b) productions from primary T cells cultured with different doses of triterpenoids and polysaccharide extracts from* G. tsugae*. Isolated CD4^+^ helper T cells (5 × 10^5^ cells/mL) from the DO11.10 transgenic mice with high percentage of OVA peptide-specific T cell receptor were cultured with different doses of Gt-TRE or Gt-PS in the presence of OVA peptide and APC for 72 hrs. The supernatants were collected and cytokine levels were determined by ELISA method. The results are expressed as the mean ± SD of four independent experiments. ^∗^ *P* < 0.05 as compared to the 0 *μ*g/mL control cells.

To investigate whether triterpenoids and polysaccharides could affect the helper T cells polarization, we isolated CD4^+^ T cells from the splenocytes of DO11.10 mice to obtain large population of T cells that can be activated by OVA peptide to induce higher antigen-specific cytokines production. The average levels of cytokines secreted from primary CD4^+^ T cells under stimulation of 1 *μ*g/mL OVA_323–339_ peptide for 72 hrs from five independent experiments were 41.4 ± 13.0 ng/mL for IL-2, 19.9 ± 11.2 ng/mL for IFN*γ*, 1.19 ± 0.10 ng/mL for IL-4, and 1.47 ± 1.29 ng/mL for IL-5. The data in Figure 3 was present as the relative cytokine production compared to the 0 *μ*g/mL control cells.

When these T cells were cultured with Gt-TRE or Gt-PS, as shown in Figure 3, the IL-4 and IL-5 productions were significantly decreased in a dose-dependent manner during T cells activation by APC and specific antigen OVA (Figure 3(a) left), but IL-2 and IFN*γ* productions were not significantly affected by Gt-TRE (Figure 3(b) left). However, the IL-4 tended to be increased by low dose of Gt-PS but significantly decreased by high doses (Figure 3(a) right), suggesting that Gt-PS exerts different effects depending on different doses. The IL-5 productions were dose dependently decreased by Gt-PS significantly decreased, reaching significantly lower at high doses. IL-2 productions were increased by Gt-PS dose dependently, reaching significantly higher at high dose of Gt-PS, whereas IFN*γ* productions were decreased by Gt-PS, especially significantly lower at higher doses of Gt-PS (Figure 3(b) right). These data suggested Gt-TRE suppressed Th2 cell polarization more than Gt-PS, whereas Gt-PS affected Th1 cell activity more than Gt-TRE.

### 3.4. Triterpenoids Suppressed but Polysaccharides Enhanced NF-*κ*B-Mediated Transcriptions

**Figure 4:**
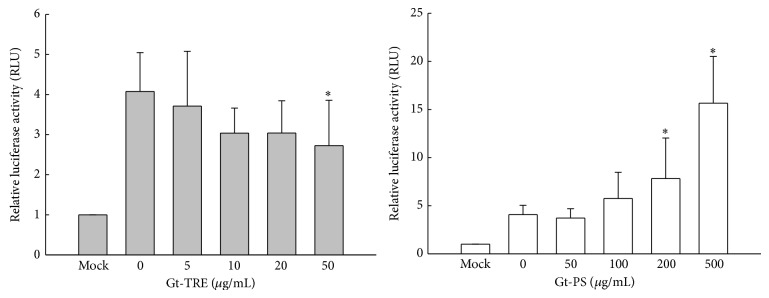
The NF-*κ*B transcriptional activity of transfected RAW264.7 cells cultured with different doses of triterpenoid and polysaccharide extracts from* G. tsugae*. RAW264.7 macrophages (1 × 10^5^ cells/mL) transiently transfected with NF-*κ*B-promoted luciferase reporter plasmid were treated with Gt-TRE or Gt-PS and stimulated with LPS (100 ng/mL) plus IFN*γ* (1000 units/mL) for 8 hr. Mocks are the RAW264.7 cells transfected with empty vector as the negative control without Gt-TRE or Gt-PS treatment. The NF-*κ*B responsive reporter luciferase activity was assayed as described in Materials and Methods. The data were representative for five independent experiments. ^∗^ *P* < 0.05 as compared to the 0 *μ*g/mL control cells.

NF-*κ*B is a converging transcription factor of various immune responses, such as cytokines expression. In order to investigate whether triterpenoids and polysaccharide may involve in NF-*κ*B-activating cytokine gene expression in macrophages and T cells, both RAW264.7 and EL-4 cells were transiently transfected with NF-*κ*B-promoted luciferase reporter plasmid. As shown in Figure 4, cells with Mock transfection showed barely reporter activity but NF-*κ*B-promoted reporter transfection had 4-fold luciferase activity under stimulation. However, NF-*κ*B-transactivation activities were gradually decreased by Gt-TRE, reaching statistical significance at the dose of 50 *μ*g/mL. On the contrary, NF-*κ*B-transactivation activities were elevated by Gt-PS in a dose-dependent manner with 16-fold luciferase activity (15.7 ± 4.9) at high dose of Gt-PS. These data suggested that polysaccharide fraction may activate macrophage through the NF-*κ*B pathway.

**Figure 5:**
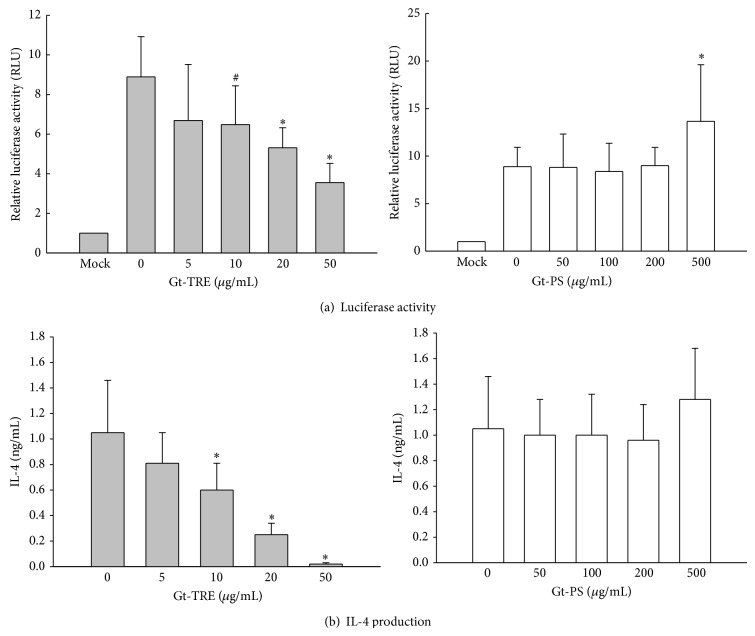
The NF-*κ*B transcriptional activity and IL-4 production by activated transfected EL4 T cells cultured with different doses of triterpenoids and polysaccharide extracts from* G. tsugae*. EL4 T cells (2 × 10^5^ cells/mL) transiently transfected with NF-*κ*B-promoted luciferase reporter plasmid were treated with Gt-TRE or Gt-PS and stimulated with 50 ng/mL PMA plus 1 *μ*g/mL ionomycin for 24 hrs. Mocks are the EL4 cells transfected with empty vector as the negative control without Gt-TRE or Gt-PS treatment. The data are representative for five independent experiments. ^#^0.05 < *P* < 0.1, ^∗^ *P* < 0.05 as compared to the 0 *μ*g/mL control cells.

When EL4 T cells were transfected with NF-*κ*B-promoted reporter plasmid, as shown in Figure 5, 9-fold luciferase activity (8.89 ± 2.03) was observed under PMA plus ionomycin-stimulation but significantly decreased in a dose-dependent manner by Gt-TRE treatment. In contrast, luciferase activity was not suppressed by Gt-PS treatment but significantly elevated to 14-fold (13.67 ± 5.96) at high dose (500 *μ*g/mL) of Gt-PS (Figure 5(a)). The cytokines secreted from transfected EL-4 cells were also measured to confirm the effects of Gt-TRE and Gt-PS (Figure 5(b)). Th2 cytokine IL-4 concentrations in Gt-TRE treated transfected EL4 T cells decreased with increased doses of Gt-TRE, which is consistent with the luciferase activity. The IL-4 levels in the Gt-PS treated were not significantly affected by Gt-PS. IL-2 productions from transfected cell were not significantly affected by Gt-TRE and Gt-PS treatments (data not shown). These data indicated that Gt-TRE decreased IL-4 production from activated-T cells, which were associated with suppression of transcription factor, NF-*κ*B, and binding activity.

## 4. Discussion

Ganoderma has been used as traditional herbal medicine in Asia, particularly in China, for thousands of years. Though much attention has been focused on the beneficial effects, recently,* Ganoderma lucidum* is the species mostly studied and published, for example, 997 publications in PubMed, compared to 45 publications for* Ganoderma tsugae* so far. There are more publications of* Ganoderma* polysaccharides than triterpenoids (394 versus 76 publications in PubMed so far). Most studies on* G. tsugae* or triterpenoids are focused on anticancer activities [36]. In this study, we evaluated the immunomodulatory effects of triterpenoid and polysaccharides fractions of* G. tsugae* on allergic responses, using macrophage and T cell lines, primary T helper cells, and NF-*κ*B-driven reporter plasmid transfected macrophage and T cells.

Our previous study demonstrated that* G. tsugae* supplementation decreased histamine release in the BALF and suppressed airway inflammatory responses in a murine model [23]. The present study showed that triterpenoid-rich extract of* G. tsugae* exerts anti-histamine release, but polysaccharide did not affect histamine release from mast cells. Triterpenoid extract possessing antihistamine effect might be the main constituent contributing to the suppression of airway inflammation observed in asthmatic mice supplemented with* G. tsugae *[23]. Histamine, an inflammatory mediator, not only plays the important role in the pathogenesis of allergic asthma, but also can modulate the Th1/Th2 responses balance [37]. Histamine upregulates Th2 cells proliferation and production of Th2 cytokines, such as IL-4, IL-5, IL-10, and IL-13, and thus drives to development of allergies [38–40]. Recent study suggested that downregulation of the Th1 responses by histamine is one of the mechanisms for Th2 environment [41].

The Th2 immune response is characterized by the development of IL-4 and IL-5-producting effector T cells, which contribute to the allergic responses in several aspects, such as eosinophilia [42]. IL-4, a pleiotropic cytokine mainly produced by activated Th2 cells, basophils, and mast cells, is an important stimulus for the switching of antibody isotype to IgE, which is often found in patients with allergic diseases [43]. Furthermore, IL-4 is crucial for eosinophils and lymphocytes recruitment to the lung and for induction of inflammation [44]. The transcription factor NF-*κ*B binds to its binding site on IL-4 promoter and upregulates IL-4 transcription [45]. Blocking NF-*κ*B activation might suppress IL-4 transcription and reduce IL-4 production and thus attenuate allergic inflammation.

Our previous study also demonstrated that* G. tsugae* supplementation modulated the Th1/Th2 balance [22]. To enhance the frequency of antigen-specific T cells in OVA-sensitized animals for better detection of antigen-specific immune responses, a detectable number of CD4^+^ T helper cells purified from DO11.10 mice were adopted in this study. Our present study demonstrated that Gt-TRE strongly decreases Th2 cytokines production not only by activated EL4 T cells but also these primary T helper cells during T cell polarization/activation. The dose-dependent decrease in IL-4 production and NF-*κ*B transactivation activity of transfected EL4 T cells confirmed the suppressive effect of triterpenoids from* G. tsugae* on IL-4 production, as shown in* in vivo* asthmatic mice supplemented with Gt-TRE [25]. This inhibition associated with NF-*κ*B pathway not only in EL4 cells but also in RAW264.7 cells suggested that triterpenoids from* G. tsugae* exert its antiallergic effects* via *downregulation of NF-*κ*B pathway not only in macrophage but also in T cells. Triterpenoid extracts, but not polysaccharide fraction, of* G. tsugae* did suppress NF-*κ*B reporter gene expression and IL-4 production after T cell activation. These data suggested that triterpenoids of* G. tsugae* exert antiallergic effects by inhibiting Th2 cell development and Th2 cytokines production and might go through downregulating NF-*κ*B transcriptional activity.

On the other hand, Gt-PS showed no effect on IL-4 secretion by activated EL-4 cells without or with NF-*κ*B transactivated reporter plasmid but tended to slightly increase IL-4 secretion by primary T helper cells at low doses and significantly decreased IL-4 and IL-5 production at high doses. This might explain the reason why eosinophils in BALF and IL-4 and IL-5 levels were significantly suppressed in OVA-sensitized and challenged allergic mice fed with Gt-TRE alone [25], but not in those fed with* G. tsugae* with both Gt-TRE and Gt-PS [23].

This study indicated that Th1 cells were not affected by triterpenoids from* G. tsugae*. The cytokines IL-2 and IFN*γ* secreted by activated primary T helper cells and EL-4 T cells were not affected by Gt-TRE. This* in vitro *study is in accordance with observations made in OVA-sensitized mice supplemented with Gt-TRE [25]. In contrast, polysaccharides of* G. lucidum* have been reported to increase T cells proliferation and IL-2 secretion [46, 47]. Our results also showed that Gt-PS enhanced IL-2 secretion in activated primary T helper cells. NF-*κ*B plays a key role on IL-2 expression during T cell development and activation [48]. The Rel A subunit of NF-*κ*B activated IL-2 transcription, whereas it suppressed IL-4 transcription [49]. It demonstrated that NF-*κ*B plays different regulatory role on Th1/Th2 cytokine expression. Other studies also demonstrated that polysaccharide of* G. lucidum* may activate dendritic cells and monocytes and promote Th1 responses* via* NF-*κ*B signal pathway [17, 18, 26]. One study on* G. lucidum* observed that polysaccharide was merely the activator for macrophage but not T lymphocytes [20]. Our study also indicated that polysaccharide of* G. tsugae* enhanced NF-*κ*B transactivation activity in both macrophage and T cells. These results suggested the different actions between Gt-PS and Gt-TRE. The more advanced study will be designed and done in the future to clarify the possible molecular mechanisms of NF-*κ*B in the Th1/Th2 regulation.

In conclusion, triterpenoid extracts of* G. tsugae* inhibited histamine release from mast cells, Th2 cytokines IL-4, and IL-5 productions in T cells and NF-*κ*B activation but did not affect Th1 cytokines IL-2 and IFN*γ* secretions. Polysaccharides of* G. tsugae* activated NF-*κ*B pathway in macrophages and enhanced IL-2 secretion in T cells but did not affect IL-4 production in T cells until high doses. These data demonstrated that triterpenoid extract is the most effective fraction of* Ganoderma tsugae* that attenuated Th2 response, which is associated with NF-*κ*B transcriptional activity.
